# Abnormal c-Fos expression in TetTag mice containing fos-EGFP

**DOI:** 10.3389/fnbeh.2024.1500794

**Published:** 2024-12-17

**Authors:** Jacob H. Wilmot, Tracy L. Warren, Cassiano R. A. F. Diniz, Deger Carda, Marrisa M. Lafreniere, Alex S. Nord, Brian J. Wiltgen

**Affiliations:** ^1^Department of Psychology, University of California, Davis, Davis, CA, United States; ^2^Center for Neuroscience, University of California, Davis, Davis, CA, United States; ^3^Department of Neurobiology, Physiology, and Behavior, University of California, Davis, Davis, CA, United States; ^4^Department of Psychiatry and Behavioral Sciences, University of California, Davis, Davis, CA, United States

**Keywords:** memory, hippocampus, mice, engram, fear

## Abstract

Molecular and genetic techniques now allow selective tagging and manipulation of the population of neurons, often referred to as “engram cells,” that were active during a specific experience. One common approach to labeling these cells is to use the *fos-tTA* transgenic mouse (TetTag). In addition to tagging cells active during learning, it is common to examine the reactivation of these cells using immediate early gene (IEG) expression as an index of neural activity. There are currently multiple TetTag lines available. The original line, cryopreserved at MMRRC, contains only the *fos-tTA* transgene, while Jackson Labs provides a version of the mouse that expresses both the *fos-tTA* and *fos-shEGFP* genes. In the current experiments, we examined IEG expression in these two mouse lines. Unexpectedly, we found that Jackson *fos-tTA/fos-shEGFP* mice express increased levels of c-Fos in the hippocampus compared to wild type animals when examined with immunohistochemistry (IHC). The expression of other IEGs, such as Arc and Egr-1, was not elevated in these mice, suggesting that the overexpression of c-Fos is not the result of increased excitability or broad changes in gene expression. qPCR revealed that Jackson *fos-tTA/fos-shEGFP* mice express mRNA corresponding to a c-Fos-Exon1-GFP fusion molecule, which may bind to C-Fos antibodies during IHC and inflate apparent c-Fos expression. Jackson *fos-tTA/fos-shEGFP* mice did not differ from their wild-type counterparts in fear expression or memory, indicating no behavioral effect of the presence of a c-Fos-GFP fusion protein. These results identify a major limitation inherent in the use of Jackson *fos-tTA/fos-shEGFP* mice.

## Introduction

A major goal of neuroscience for more than a century has been to identify and manipulate the memory engram: the set of physical and chemical changes that underlie the storage of a specific memory in the brain (Josselyn et al., 2015). One major area of interest has been to identify the population of cells, often referred to as “engram cells,” in which these changes occur. The activity of these neurons during experience is thought to be responsible for encoding memories and their reactivation drives memory retrieval (Liu et al., 2012; Tanaka et al., 2014).

Early research using single-unit recordings led to the discovery of place cells in the hippocampus, which have many properties consistent with memory encoding and storage (O’Keefe and Dostrovsky, 1971). For example, place cells exhibit highly stable and environment-specific coding of the animal’s location in space (Muller and Kubie, 1987; Thompson and Best, 1989). Moreover, the formation and retention of place fields requires cellular mechanisms known to be important for learning and memory (e.g., NMDA receptor activation, protein synthesis) (Rotenberg et al., 1996; Kentros et al., 1998; Agnihotri et al., 2004). Subsequent research revealed that learning in a novel environment induces the expression of immediate early genes (IEGs) like c-Fos, Arc, and Zif268 in a subpopulation of hippocampal neurons (Guzowski et al., 1999, 2001). The expression of IEGs in these cells exhibits properties remarkably consistent with both place cell activity and memory encoding/storage. Similar percentages of cells in each subregion of the hippocampus display place fields and express IEGs, IEG expression is environment specific, and the same cellular mechanisms required for place cell retention and memory encoding are also required for IEG expression (Kubik et al., 2007). Because of these properties, IEGs have since been used to identify putative engram cells (Reijmers et al., 2007; Tayler et al., 2013).

The use of IEGs as a marker of memory encoding cells allowed researchers to develop genetic methods to target these cells for manipulation with modern techniques such as opto- and chemogenetics. One of the earliest and most popular methods for tagging these cells is the fos-tTA (or TetTag) transgenic mouse (Reijmers et al., 2007). In these mice, activation of the c-Fos promoter induces the expression of the tetracycline transactivator (tTA). tTA can then be used to drive the expression of a desired effector protein such as a flurorescent marker, opsin, or DREADD (designer receptors activated by designer drugs), under the control of the tetracycline-inducible promoter (tetO). Tetracycline/doxycyline suppresses this system by preventing tTA from binding to the tetO promoter. This allows temporal control over the tagging window via the inclusion or exclusion of doxycycline in the animals’ diet, thereby permitting the tagging of cells active during specific experiences.

Shortly after their development, TetTag mice were used to show that cells that express c-Fos during memory encoding are reactivated during memory retrieval (Tayler et al., 2013). Additionally, this reactivation is both necessary and sufficient for memory retrieval; optogenetic inhibition of these c-Fos + cells prevents memory retrieval (Tanaka et al., 2014), while their activation can induce retrieval (Liu et al., 2012). Following these discoveries, substantial work has been dedicated to characterizing the cellular and physiological properties of engram cells. For example, TetTag mice have been used to study synaptic changes and connectivity between memory encoding cells (Ryan et al., 2015; Abdou et al., 2018), learning-induced changes in intrinsic excitability and metaplasticity (Crestani et al., 2019), and the neurobiology underlying memory deficits caused by disease (Roy et al., 2016). In addition to studying the properties of memory encoding cells, many studies have used TetTag mice to seek answers to longstanding questions in neuroscience by determining whether artificial activation of these cells can be used to drive various neural and behavioral processes including the creation of false memories (Garner et al., 2012; Ramirez et al., 2013), linking previously unrelated memories (Ohkawa et al., 2015), and systems consolidation of memory (Kitamura et al., 2017; de Sousa et al., 2019), as well as some phenomena outside the field of memory (Zhang et al., 2015).

Studies using the TetTag mouse to label memory encoding cells often also utilize endogenous IEG expression to quantify neural activity sometime after the period of tagging. For example, endogenous c-Fos expression during memory retrieval may be used to examine the reactivation of cells that were tagged during memory encoding (Reijmers et al., 2007; Liu et al., 2012; Tayler et al., 2013; Tanaka et al., 2014). However, to the best of our knowledge, IEG expression in TetTag animals has never been carefully examined in comparison to non-transgenic mice. Given the widespread use of IEG quantification in TetTag mice, we set out to thoroughly characterize the endogenous expression of IEGs in these animals and compare it to their wild type littermates.

Currently, at least two variants of the fos-tTA transgenic mouse are available. The original line of mice (*fos-tTA* mice) is cryopreserved at the NIH’s Mutant Mouse Resource and Research Centers (MMRRC), while Jackson Labs carries a different line that contains both the *fos-tTA* and the *fos-shEGFP* transgenes (*fos-tTA/fos-shGFP* mice). Here, we examine IEG expression in each transgenic line.

## Results

### Expression of tTA and sh-GFP in the MMRRC and Jackson TetTag lines

**FIGURE 1:**
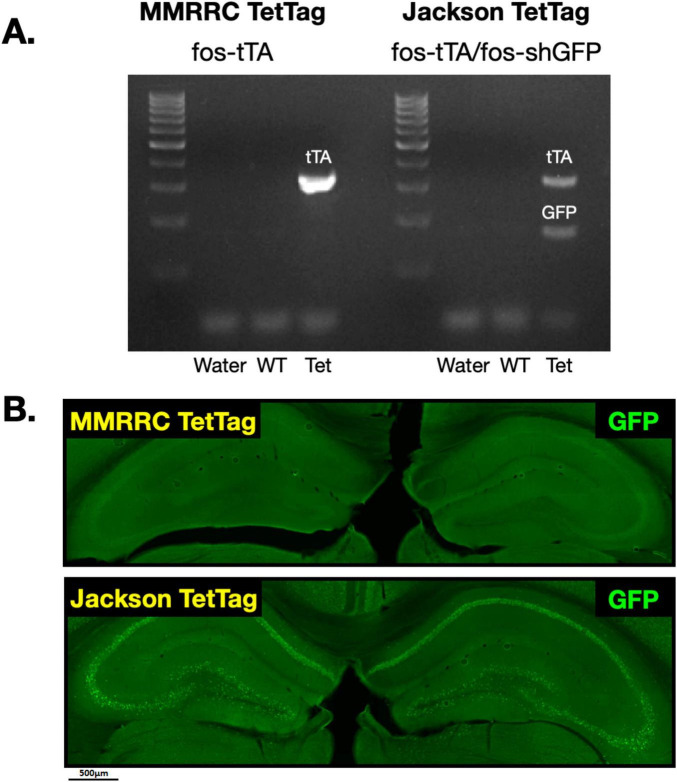
GFP expression in MMRRC *fos-tTA* and Jackson *fos-tTA/fos-shGFP* TetTag mice. **(A)** Example of genotyping for *fos-tTA* and *fos-shGFP* in the Jackson and MMRRC mice. The *GFP* gene was present in Jackson *fos-tTA/shGFP mice*, but not MMRRC *fos-tTA* mice. **(B)** Example images of GFP IHC in Jackson *fos-tTA/shGFP mice* and *fos-tTA* MMRRC mice. GFP was present in Jackson *fos-tTA/shGFP*, but not MMRRC *fos-tTA* mice.

We first verified that both MMRRC and Jackson TetTag mice express tTA, while GFP is only found in the latter line. To do this we performed PCR (Figure 1). In MMRRC transgenic mice, but not their wildtype (WT) littermates, we observed a band corresponding to tTA but not GFP. In Jackson transgenic mice, there was a band for tTA and GFP that was not found in their littermate controls (Figure 1A). Next, we fear conditioned mice from each line and stained brain slices for GFP. As expected, GFP positive neurons were observed throughout the dorsal hippocampus in the Jackson TetTag line but not in MMRRC animals (Figure 1B). Interestingly, the number of GFP positive neurons in the Jackson mice appeared to be much higher than the levels of endogenous c-Fos expression we typically observe after context fear conditioning (Tanaka et al., 2014; Nakazawa et al., 2016). We therefore conducted a series of experiments comparing c-Fos expression in these two lines.

### Immunohistochemistry detects more C-Fos + neurons in Jackson fos-tTA/fos-shGFP transgenic mice

**FIGURE 2:**
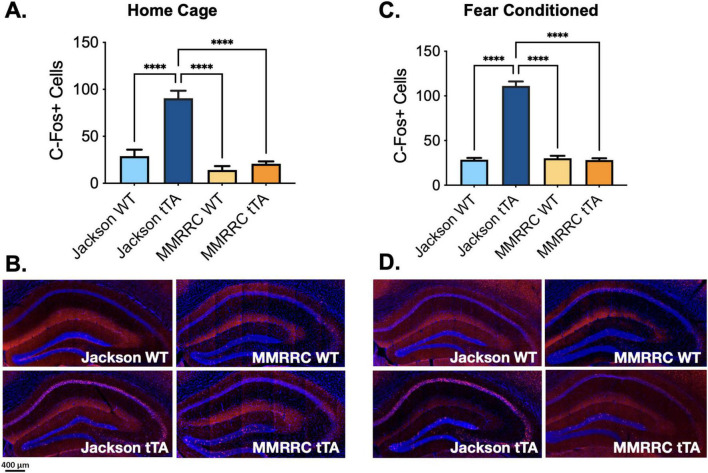
C-Fos immunohistochemistry reveals higher C-Fos expression in Jackson *fos-tTA/fos-shGFP* versus MMRRC *fos-tTA* TetTag mice. **(A)** The number of c-Fos + cells was higher in naive homecage Jackson *fos-tTA/fos-shGFP* mice (*n* = 11) compared to their wildtype littermates (*n* = 8) and MMRRC wildtype (*n* = 4) or tTA mice (*n* = 4). **(B)** Examples of c-Fos IHC staining in home cage mice. **(C)** The number of c-Fos + cells was higher in fear conditioned Jackson *fos-tTA/fos-shGFP* mice (*n* = 11) compared to their wildtype littermates (*n* = 4) and MMRRC wildtype (*n* = 5) or *fos-tTA* mice (*n* = 5). **(D)** Examples of C-Fos IHC staining in fear conditioned mice. *⁣*⁣**p < 0.0001.

Having determined that Jackson *fos-tTA/shGFP* mice may express a fusion molecule of c-Fos and GFP, we next evaluated whether the presence of this molecule impacts c-Fos expression as measured with immunohistochemistry (IHC) by staining for c-Fos in Jackson *fos-tTA/shGFP* and MMRRC *fos-tTA* mice. To determine whether the transgenic mice express c-Fos at levels similar to their wild type litter mates, we used IHC to examine endogenous c-Fos expression in the dorsal dentate gyrus in behaviorally naïve home-cage mice. Dentate gyrus was chosen because it is a commonly studied area in “engram” studies. Unexpectedly, there were more c-Fos + neurons in the Jackson *fos-tTA/shGFP* mice than in MMRRC *fos-tTA* mice or wild type mice from either strain (*F*(3, 23) = 23.1, *p* < 0.0001; all *post hoc* comparisons versus Jackson *fos-tTA/shGFP, p* < 0.0001; Figure 2A). We also examined c-Fos expression after contextual fear memory retrieval and found similar results; there were more c-Fos + neurons in the Jackson *fos-tTA/shGFP* mice than in any other group (*F*(3,21) = 99.4, *p* < 0.0001; all *post hoc* comparisons versus Jackson *fos-tTA/shGFP*, *p* < 0.0001; Figure 2C). Comparison of c-Fos between home cage and fear conditioned animals revealed significant effects of fear conditioning (*F*(1, 45) = 4.4, *p* < 0.05) and genotype (*F*(3, 45) = 83.8, *p* < 0.001), but no interaction (*F* (3, 45 = 1.1, *p* > 0.05). Although we quantified c-Fos expression in the dentate gyrus, the effect is also qualitatively apparent in other hippocampal subregions like CA1 (Figures 2B, D).

### Arc and Zif268 are not overexpressed in Jackson fos-tTA/fos-shGFP mice

**FIGURE 3:**
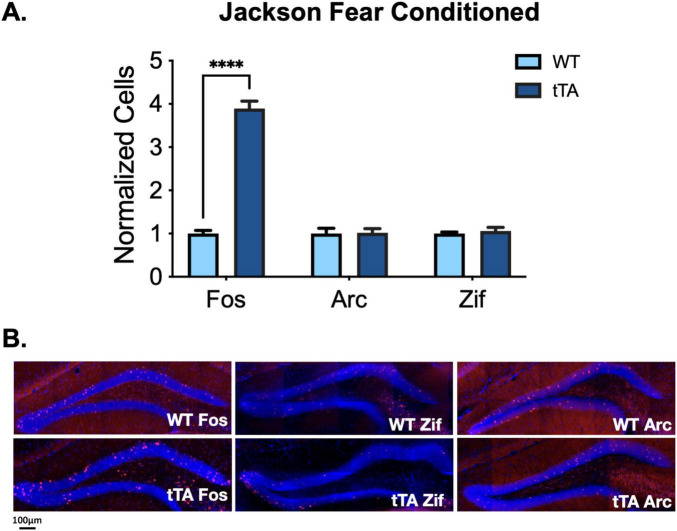
IEG expression in Jackson *fos-tTA/fos-shGFP* mice. **(A)** Arc and Zif268 expression are unaltered in Jackson *fos-tTA/fos-shGFP* transgenic mice (*n* = 4) compared to their wild type littermates (*n* = 5), while C-Fos expression is elevated. C-Fos data is repeated from Figure 2C. **(B)** Example images of C-Fos, Zif268, and Arc IHC staining in Jackson *fos-tTA/fos-shGFP* mice and wildtype mice from the same colony. *⁣*⁣**p < 0.0001.

To determine if the apparent overexpression of c-Fos, as measured by IHC, is the result of hyperexcitability or global increases in IEG expression, we quantified Zif268 and Arc expression in the dorsal DG of Jackson *fos-tTA/fos-shGFP* mice and wild-type animals from the same colony after contextual fear conditioning. After normalizing for the amount of stained cells in wild-type animals for each IEG, we found a difference only in the number of c-Fos + cells between transgenic and wild-type animals, as reported above (genotype X IEG interaction: *F*(2, 27) = 50.6, *p* < 0.0001; Figures 3A, B). We found no differences in the number of Zif268 + cells (*t*(27) = 0.25, *p* > 0.99) or Arc + cells (*t*(27) = 0.07, *p* > 0.99), suggesting the effect observed for c-Fos is not the result of increased neural activity or broad changes in expression of IEGs.

### Jackson fos-tTA/fos-shGFP mice express mRNA for a C-Fos-Exon1-GFP fusion molecule

**FIGURE 4:**
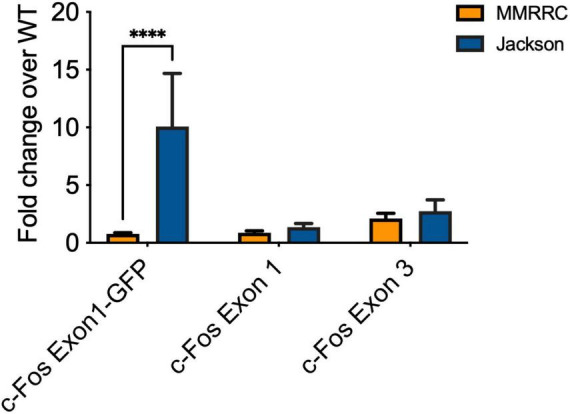
qPCR reveals the presence of C-Fos Exon1-GFP fusion mRNA in Jackson *fos-tTA/fos-shGFP* (*n* = 3) but not MMRRC *fos-tTA* mice (*n* = 7). *⁣*⁣**p < 0.0001.

Given that elevated c-Fos expression is not observed in the MMRRC TetTag transgenic line, we suspected it may be related to the *fos-EGFP* transgene that is only expressed in the Jackson line. Previous papers have reported that this transgene contains the c-Fos promoter as well as several exons/introns from the endogenous c-Fos gene (Kim et al., 2015). If this produces a c-Fos-EGFP fusion protein it could be detected by our c-Fos antibody. The detection of both endogenous c-Fos and the c-Fos-EGFP protein could explain why levels of this immediate early gene, but not others, appear to be elevated. Therefore we tested for the expression of a fused cFos-Exon1-GFP RNA molecule in Jackson *fos-tTA*/f*os-shGFP* mice and mice from the MMRRC *fos-tTA* line (Figure 4). qPCR showed an increase in detection of such a molecule in the Jackson *fos-tTA/fos-shGFP* mice, but not in mice from the MMRRC *fos-tTA* line (*p* < 0.05). By contrast, qPCR for RNA corresponding to cFos-Exon1 and cFos-Exon3 showed no difference between the two mouse lines (respectively, *p* = 0.167 and *p* = 0.548). This indicates that mRNA corresponding to a fused cFos-Exon1-GFP molecule is present in the Jackson *fos-tTA/fos-shGFP* mice but not the MMRRC *fos-tTA* mice.

### Contextual fear memory is not different between Jackson wild type and fos-tTA/fos-shGFP transgenic mice

**FIGURE 5:**
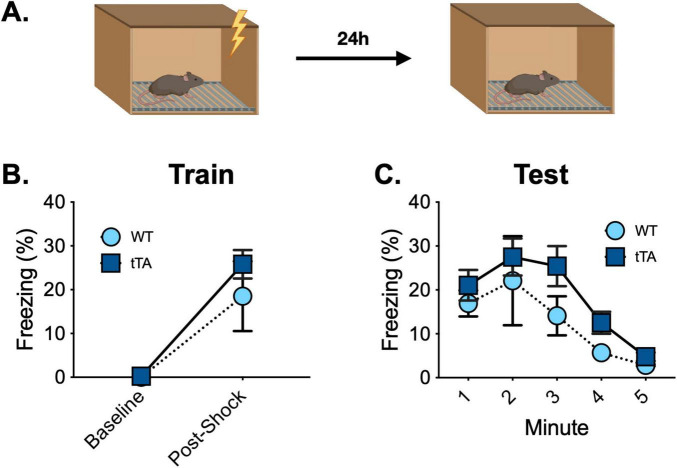
Fear conditioning behavior is similar between Jackson *fos-tTA/fos-shGFP* mice (*n* = 12) and their wildtype littermates (*n* = 12). **(A)** Mice underwent contextual fear conditioning and were tested for context fear 48 h later. **(B)** Freezing increased after foot shocks similarly across genotypes. **(C)** Contextual fear memory retrieval was similar across genotypes.

To determine if there are any functional behavioral consequences of the presence of the c-Fos -Exon1-GFP molecule, we examined memory acquisition and retrieval in Jackson wild type (*n* = 4) and *fos-tTA*/*fos-shGFP* (*n* = 4) animals. The mice underwent contextual fear conditioning and received a 5-min memory test 2 days later (Figure 5A). As expected, freezing increased after the shocks during training, but there were no differences in baseline or post-shock freezing between groups (Main effect of time *F*(1, 14) = 37.8, *p* < 0.0001; No effect of genotype, *F*(1, 14) = 1.1, *p* > 0.05; No time x genotype interaction, *F*(1, 14) = 0.96, *p* > 0.05) (Figure 5B). Similarly, freezing decreased across time during the memory test, but no differences were observed between groups [Main effect of time *F*(4, 56) = 12.01, *p* < 0.0001; No effect of genotype, *F*(1, 14) = 1.44, *p* > 0.05; No time × genotype interaction, *F*(4, 56) = 0.50, *p* > 0.05] (Figure 5C). Thus, the acquisition and retrieval of contextual fear memory is unaltered in Jackson *fos-tTA/fos-shEGFP* transgenic mice compared to wild type animals.

## Discussion

Here, we characterized the expression of c-Fos in Jackson *fos-tTA/fos-shEGFP* and MMRRC *fos-tTA* transgenic mice. Our results suggest that Jackson *fos-tTA/fos-shEGFP* express a C-Fos-Exon1-GFP fusion protein which may bind to c-Fos antibodies during IHC, resulting in the apparent overexpression of c-Fos when compared to their wild type littermates. c-Fos appears overexpressed in IHC experiments in both behaviorally naïve and fear conditioned mice. However, two other IEGs (Arc and Zif268), are expressed normally in these animals, indicating that the elevated c-Fos is not the result of hyperexcitability or widespread changes in gene expression.

Exon 1 of the *c-Fos* gene encodes the N-terminus of the c-Fos protein – a region which many of the commercially available c-Fos antibodies detect. Therefore, we hypothesize that c-Fos expression appears elevated in these mice, when examined using IHC, because N-terminus antibodies detect both endogenous c-Fos protein and the shEGFP protein. Detection of both proteins would be expected to produce a stronger signal even in home-cage conditions where endogenous c-Fos expression is low, but not absent. While some antibodies exist that bind to the C-terminus of the c-Fos protein, we were not able to obtain usable IHC images with these antibodies to further test this hypothesis. Future studies could use *in situ* hybridization to examine c-Fos RNA using probes that do not detect the N-terminus sequence (Exon 1). At present, we recommend that researchers using Jackson *fos-tTA/fos-shGFP* mice use Arc or Zif268 as alternatives to c-Fos in studies using IEGs as a marker of activated neurons.

Given that only a portion of the c-Fos gene is overexpressed and is likely fused to a GFP molecule, the apparent elevation of c-Fos expression is unlikely to produce functional consequences in the affected neurons, as evidenced by the lack of behavioral abnormalities in these mice. However, this alteration in the detection of c-Fos by immunohistochemistry (IHC) does diminish the utility of these animals for studies that use c-Fos IHC staining as a measure of neuronal activation or reactivation. Because c-Fos staining is very high in the hippocampus of these mice even in home-cage conditions, when endogenous c-Fos is known to be expressed at very low levels, the use of c-Fos IHC to measure neuronal activity in these animals would not be advised. To avoid this issue, we recommend the use of the *fos-tTA* mouse that does not contain the *fos-shEGFP* transgene, which is available from the Mutant Mouse Resource and Research Centers (031756-MU, MMRRC). These animals have also been used by several labs and there does not appear to be excessive c-Fos labeling in these mice, though there are not extensive images available (Okuyama et al., 2016; Abdou et al., 2018). Alternatively, if brain wide labeling is not necessary, it is possible to use a viral strategy of co-injecting a *fos-tTA* AAV and an AAV containing the desired effector gene under the control of the tetracycline response element (TRE), as several recent publications have demonstrated (Roy et al., 2016; Chen et al., 2019).

The over-labeling of c-Fos is a major drawback to the use of the Jackson *fos-tTA/fos-shEGFP* transgenic mouse – a popular strategy for activity-dependent neuronal tagging. However, numerous other transgenic and viral IEG-dependent tagging methods have also been developed recently (Guenthner et al., 2013; Cazzulino et al., 2015; Sørensen et al., 2016). With this surge in studies using activity-dependent labeling strategies to observe and manipulate the neuronal ensembles underlying specific memories, sometimes referred to as “engrams,” it is important that the methods used to perform these experiments are fully vetted, so that their results can be interpreted in meaningful ways.

## Materials and methods

### Subjects

Subjects in this study were 62 (28 male and 34 female) triple transgenic *fos-tTA/fos-shEGFP/tetO-H2B-GFP* (TetTag) or double transgenic *fos-tTA/fos-shEGFP* mice. The generation of these mice has been described previously (Reijmers et al., 2007; Tayler et al., 2013). Briefly, *fos-tTA/fos-shGFP* mice (#018306, Jackson Laboratory) were crossed with *tetO-H2B-GFP* mice (#005104, Jackson Laboratory). The resulting animals express H2B-GFP under the *tetO* promoter and the tetracycline-transactivator (tTA) protein under the *c-fos* promoter. The H2B-GFP transgene was not utilized in this study, and we found no differences between H2B-GFP + and H2B-GFP- mice. No sex differences in C-Fos expression were observed; therefore, male and female mice were pooled for analyses. All mice were then maintained on a C57BL/6J background. Control, wild type animals were mice from the same colony, but negative for the transgenes. *fos-tTA* mice without the *fos-shEGFP* transgene were a gift from Dr. Mark Mayford and were treated the same as all other mice. The mice were raised on a diet of chow incorporated with low concentrations of doxycycline (DOX chow, 40 mg/kg, Envigo). Mice were on a 12 h light/dark cycle (lights on at 7am) and had *ad libitum* access to food and water throughout the experiment. At 21 days, pups were weaned and small tail clippings were collected for genotyping. All mice were 2–4 months old at the beginning of experimental manipulations. Two weeks before the experiment began, mice were single housed and allowed to acclimate to the within-laboratory vivarium. All experiments were conducted in accordance with the UC Davis Institutional Animal Care and Use Committee (IACUC).

### Genotyping

gDNA of each mouse in the colony was isolated from tail clips using the Zymo Research Miniprep Plus Kit (Zymo Research #D4068) following the manufacturer’s protocol. DNA samples previously identified as either wild-type or fos-tTA positive from both the MMRRC TetTag and the Jackson TetTag lines, or water control, were added to a PCR mix of GoTaq Hot Start Green Master Mix (Promega #M5122), Nuclease-Free water, fos-tTA (forward: ACCTGGACATGCTGTGATAA, reverse: TGCTCCCATTCATCAGTTCC) and fos-EGFP (forward: AAGTTCATCTGCACCACCG, reverse: TCCTTGAAGAAGATGGTGCG) primers. Resulting PCR samples were amplified with a GoTaq Hot Start PCR cycle. A 4% Agarose gel with Ethidium Bromide was prepared and PCR products were loaded onto the gel alongside a 1 Kb DNA ladder. DNA fragments were separated for 30 min by gel electrophoresis powered to 130 V and imaged by exposing to UV light.

### Apparatus

The behavioral apparatus has been described previously (Tayler et al., 2011; Wilmot et al., 2019). Briefly, contextual fear conditioning occurred in a conditioning chamber (30.5 cm × 24.1 cm × 21.0 cm) within a sound-attenuating box (Med Associates). The chamber consisted of a front-mounted scanning charge-coupled device video camera, stainless steel grid floor, a stainless steel drop pan, and overhead LED lighting capable of providing broad spectrum or infrared light. The conditioning chamber was lit with both broad spectrum and infrared light, scented with 70% ethanol, and background noise was generated with a fan in the chamber and HEPA filter in the room. The chamber was cleaned with 70% ethanol before each session.

### Behavioral procedure

All behavioral procedures occurred during the light portion of the light/dark cycle. All animals were habituated to handling for 5 min/day for 5 days prior to the beginning of behavioral experiments. Off DOX mice were taken off doxycycline for 48 h before training, while On DOX mice remained on doxycycline chow for the duration of the experiment. Mice in the fear conditioning groups were trained at the end of this 48h period. Training consisted of a 3 min baseline period of context exposure followed by 4 foot shocks (2 s, 0.75 mA) separated by a 1 min intertrial interval. Mice were removed from the chamber 1 min after the last shock. Immediately following training, Off DOX mice were given high concentration DOX chow (1 g/kg) for 24 h to rapidly suppress tTA and then remained on low concentration DOX for the rest of the study. No differences were found between On DOX and Off DOX mice, so these groups were pooled for analyses. Mice were returned to the conditioning chamber 2 days after training for a 5 min memory test. Freezing behavior was measured automatically using VideoFreeze (Med Associates). Home cage mice remained in their home cage for the duration of the experiment until they were sacrificed.

### qPCR

Three mice from the TetTag line, three wild type mice, and seven mice from the Mayford line were randomly selected for qPCR experiments. Mouse cortex was collected and immediately dissected into RNAlater and stored at −20°C. RNA was subsequently extracted using the Qiagen RNeasy kit (Qiagen #74104) following the manufacturer’s protocol. RNA was reverse transcribed into cDNA using the Qiagen QuantiTect Reverse Transcription Kit (Qiagen #205311) following the manufacturer’s protocol. qPCR was conducted on cDNA using Applied Biosystems Power SYBR Green Master Mix (Thermo Fisher #4367659) and the following primers:

**Table:** 

Amplified RNA molecule	Forward primer	Reverse primer
cFos_Exon1	GGGTTTCAAC GCCGACTA	GCTGGGGAATG GTAGTAGGAA
cFos_Exon1-GFP hybrid	GGGTTTCAA CGCCGACTA	GGTGTTCTG CTGGTAGTGGT
cFos_Exon3	TGGAGCCAG TCAAGAGCATC	AACCGGAC AGGTCCACATCT
GAPDH	TCACCACCA TGGAGAAGGC	GCTAAGCAG TTGGTGGTGCA

Three technical qPCR replicates were used for each mouse/RNA molecule combination. The range of cycle counts across technical replicates was calculated for each condition. If the range was higher than two, the technical replicate that was furthest from the other two replicates was excluded. Following this, the range of cycle counts for technical replicates within each condition was less than or equal to two, and all conditions had at least two technical replicates remaining. Expression levels were calculated for each RNA molecule of interest using the 2^–ΔΔCt^ method (Livak and Schmittgen, 2001) and using GAPDH as a housekeeping gene. First, the mean cycle count for GAPDH for each mouse was subtracted from the mean cycle count for the mouse/condition of interest (Δ Ct). Then, the mean Δ Ct for wild type mice was subtracted from the ΔCt for each Mayford or TetTag mouse/condition (ΔΔ Ct). Finally, fold changes of RNA molecule expression over that observed in wild type mouse were calculated by raising 2 to the power of −ΔΔCt for each Mayford or TetTag mouse/condition (2^–ΔΔCt^).

### Immunohistochemistry

Ninety minutes after behavioral testing, mice were transcardially perfused with 4% PFA. Following 24 h of post-fixation, 40 μm coronal sections were cut and stained for c-Fos. Slices were washed three times in 1X phosphate buffered saline (PBS) at the beginning of the procedure and after all antibody and counterstaining steps. All antibodies and counterstains were diluted in a blocking solution containing.2% Triton-X and 2% normal donkey serum in 1X PBS, unless otherwise indicated. First, sections were incubated for 15 min in the blocking solution. Then, slices were incubated for 24 h at four degrees in anti-c-Fos (1:5000, rabbit, ABE457, Millipore), anti-Arc (1:1000, rabbit, 156 002, Synaptic Systems), anti-EGR1 (1:750, rabbit, 4153, Cell Signaling) or anti-GFP (1:5000, 13970, Abcam) primary antibodies. Next, slices were placed in biotinylated donkey anti-rabbit or anti-chicken secondary antibody (1:500, Jackson ImmunoResearch) for 1 h at room temperature, followed by Streptavidin-Cy2 or Cy3 (1:500, Jackson ImmunoResearch) for 45 min. Finally, sections were stained with DAPI (1:10,000 in PBS, Life Technologies) for 10 min, mounted on slides, and coverslipped with Vectashield anti-fade mounting media (Vector Labs).

### Image acquisition and quantification

Images were acquired at 20× magnification using a fluorescence slide scanner (BX61VS, Olympus), then cropped to include the dorsal dentate gyrus. A blinded experimenter performed cell counts on the entire suprapyramidal blade of the dentate gyrus in 3–4 sections from each animal (6–8 hemispheres). Cells were counted using the multi-point tool in Image-J. Counts were averaged across slices to obtain one value per animal.

### Statistical analyses

Behavioral data was analyzed using repeated-measures ANOVA. For the training session, baseline freezing is the average freezing during the 3 min of context exposure prior to the first shock. Post-shock freezing is the freezing averaged across all 1-min interstimulus intervals between shocks. H2B-GFP cell count data were analyzed using unpaired *t*-tests. IEG cell count data was analyzed using ANOVA followed by Bonferroni *post hoc* comparisons as necessary. The Mann-Whitney U test was used to test for differences between mouse lines in qPCR RNA expression data for each molecule of interest.

## Data Availability

The original contributions presented in this study are included in this article/supplementary material, further inquiries can be directed to the corresponding author.
